# Tryptophan Alleviates Acute Heat Stress-Induced Impairment of Antioxidant Status and Mitochondrial Function in Broilers

**DOI:** 10.3389/fvets.2022.863156

**Published:** 2022-03-31

**Authors:** Jingxin Ouyang, Hua Zhou, Qiufen Li, Jun Zheng, Chun Chen, Shuaipeng Guo, Jinming You, Guanhong Li

**Affiliations:** ^1^Jiangxi Province Key Laboratory of Animal Nutrition, College of Animal Science and Technology, Jiangxi Agricultural University, Nanchang, China; ^2^Jiangxi Province Key Innovation Center of Integration in Production and Education for High-Quality and Safe Livestock and Poultry, Nanchang, China

**Keywords:** broiler, heat stress, antioxidant capacity, mitochondrial function, tryptophan

## Abstract

Heat stress has been considered as a critical risk factor for decreasing performance and causing oxidative stress in broilers. The tryptophan (TRP) derivative 5-hydroxytryptophan has been reported to protect membrane fluidity in broilers suffering from oxidative stress. Therefore, this experiment was conducted to investigate the effects of dietary TRP supplementation on antioxidant status and mitochondrial function-related genes expressions in broilers exposed to acute heat stress (34 ± 1°C, 24 h). Female Arbor Acres broilers (19-d-old, *n* = 180) were randomly assigned to 1 of 3 treatments. Broilers were fed a basal diet and in the thermoneutral conditions (TN, 23 ± 1°C) was considered as the TN group. Broilers were fed a basal diet and exposed to acute heat stress (HS, 34 ± 1°C) was regarded as the HS group. Broilers were fed a basal diet supplemented with 0.18% L-tryptophan and under HS conditions was treated as the HS + TRP groups. Heat stress led to increased malondialdehyde (MDA) concentration (*P* < 0.05), while it elevated catalase (CAT), glutathione peroxidase (GSH-Px), superoxide dismutase (SOD), and total antioxidant capacity activities (T-AOC) (*P* < 0.05) compared with the TN group. Nevertheless, compared with the HS group, TRP supplementation increased SOD activity (*P* < 0.05). The effects of acute heat stress were associated with increased mRNA abundance for redox-related genes (*P* < 0.05), and reduced mRNA levels for mitochondrial function-related genes (*P* < 0.05). Notably, the effects of acute heat stress on mitochondrial function-related genes expressions were reversed by TRP treatment. Collectively, dietary 0.18% TRP supplementation beneficially protects against acute heat stress-induced oxidation stress and mitochondrial dysfunction by regulating antioxidant states and increasing mitochondrial function-related genes expressions in broilers.

## Introduction

Heat stress (HS) has been known to adversely affect the growth rates, feed intake, and meat quality in animals (1, 2). Because of lots of feathers and a lack of sweat glands on the skin, poultry is particularly susceptible to HS (3). Meanwhile, HS can increase reactive oxygen species (ROSs) production and cause oxidative stress in broilers (4–8). Deterioration of the broilers' metabolic, intestinal health, immune and endocrine functions are the most common complications related to HS exposure and oxidative stress (8–10). Notably, mitochondrial dysfunction is closely related to oxidative stress (11–13). Taken together, it is urgent to develop an effective strategy to lighten the damage of HS on broilers. Nutritional intervention is a promising strategy to mitigate the detrimental effects of stress and to improve performance and nutrition utilization (14). Supplementation with lecithin or *Bacillus subtilis* has been demonstrated to improve the growth performance and meat quality of broilers (15, 16). Tryptophan (TRP) has been considered to be a functional amino acid, which is important for maintenance, reproduction, and immunity (17). Meanwhile, an increase in the dietary TRP has been shown to improve the daily gain, feed utilization ratio, and immune function (18–20), and also alleviate oxidative stress and chronic unpredictable stress in broilers (21, 22). Moreover, TRP has been reported to enhance antioxidant capacity, intestinal integrity, and mitochondrial function in diquat-stressed piglets (23). However, whether TRP supplementation can improve the antioxidant capacity and mitochondrial function in broilers reared under acute heat stress is largely unknown. Therefore, this study was conducted to determine the effects of dietary TRP supplementation on antioxidant capacity and mitochondrial function in broilers subjected to acute heat stress.

## Materials and Methods

### Ethical Approval

Experimental procedures used in this study were performed following the Laboratory Animal Welfare and Ethics Censorship and approved by the Laboratory Animals Ethics Committee of Jiangxi Agricultural University (Nanchang, Jiangxi, China).

### Animals and Experimental Design

A total of 180 1-day-old female Arbor Acres broilers were reared in a temperature and light-control house and fed a starter diet for 18 days under a standard management program. The chicks were under continuous light for 1 week and then changed to natural lighting. The chicks were vaccinated routinely. At 19 d of age, broilers with similar body weight (481.50 ± 26.68 g) were allocated to the three dietary treatments with six replicate pens per treatment and 10 broilers per pen. Broilers in the thermoneutral conditions (TN) group were fed the basal diet [which was formulated according to the requirements of NRC (1994), Table 1] and housed in a temperature-controlled room. Broilers in the acute heat stress (HS) and HS + TRP groups were housed in another temperature-controlled room and were fed the basal diet and the basal diet supplemented with 0.18% TRP, respectively. At 21 d of age, broilers in the HS and HS + TRP groups were exposed to acute heat stress (34 ± 1°C, relative humidity, 65–70%) for 24 h, and broilers in the TN group were continuously kept in the TN environment (23 ± 1°C, relative humidity, 65–70%). L-Glutamate was added to the basal diet instead of L-TRP to make all diets isonitrogenous. Broilers were allowed *ad libitum* access to feed and water.

**Table 1:** Ingredients and nutrient compositions of experimental diets.

**Ingredients (%)**	**Starter**	**TN**	**HS**	**HS+ TRP**
Corn	52.50	57.00	57.00	57.00
Soybean meal, 43% CP	23.00	16.20	16.20	16.20
Corn gluten meal, 64% CP	10.00	10.00	10.00	10.00
Extruded soybean, 34% CP	6.00	8.00	8.00	8.00
Soybean oil	2.50	3.50	3.50	3.50
Limestone	1.50	1.50	1.50	1.50
Dicalcium phosphate	1.70	1.50	1.50	1.50
Mineral premix^a^	0.20	0.20	0.20	0.20
Compound Vitamin premix^b^	0.03	0.03	0.03	0.03
NaCl	0.30	0.30	0.30	0.30
L-Lysine HCl (79%)	0.20	0.31	0.31	0.31
Methionine (98%)	0.15	0.10	0.10	0.10
Tryptophan (99%)	0.00	0.00	0.00	0.18
Glutamic acid	0.00	0.39	0.39	0.13
Choline chloride	0.10	0.10	0.10	0.10
Zeolite powder	1.82	0.87	0.87	0.95
Total	100.00	100.00	100.00	100.00
**Calculated nutritional content**				
Metabolizable energy (MJ/kg)	12.81	13.40	13.40	13.40
Crude protein (%)	22.38	20.48	20.48	20.48
Calcium (%)	1.05	0.99	0.99	0.99
Available phosphorus (%)	0.46	0.41	0.41	0.41
Tryptophan (%)^c^	0.23	0.20 (0.18)	0.20 (0.18)	0.38 (0.34)
Lysine (%)	1.16	1.11	1.11	1.11
Methionine (%)	0.56	0.48	0.48	0.48

a *The mineral premix provided the following per kg of the diet: Cu (Cu_2_ (OH)_3_Cl), 8 mg; Mn (MnSO_4_·H_2_O), 60 mg; Se (Na_2_SeO_3_), 0.15 mg; Fe (FeSO_4_·H_2_O), 80 mg; Zn (ZnSO_4_·H_2_O), 40 mg; I (Ca (IO_3_)_2_), 0.35 mg*.

b *The vitamin premix provided the following per kg of the diet: vitamin A, 12,000 IU; vitamin D_3_, 3,000 IU; vitamin E, 36 IU; vitamin K_3_, 0.90 mg; vitamin B_1_, 0.60 mg; vitamin B_2_, 2.40 mg; vitamin B_6_, 1.80 mg; vitamin B_12_, 0.015 mg; D-biotin, 0.18 mg; D-pantothenic acid, 3 mg; Folic acid, 0.75 mg; Niacinamide, 38 mg*.

c *Data in parentheses indicate the analyzed values*.

### Sample Collection and Preparation

After heat stress for 24 h, one broiler per replicate was randomly selected and slaughtered for sample collection. Blood samples were collected from the wing vein and were centrifuged at 1,000 g for 10 min at 4°C to obtain serum, and stored at −20°C for further analysis. Then, the selected broilers were euthanized by cervical dislocation. The ileum mucosa was collected by scraping the intestinal wall with a glass microscope slide, immediately frozen in liquid nitrogen, and stored at −80°C until the analysis of the antioxidant capacity. This was followed by collecting liver samples, immediately frozen at −80°C until analysis of mitochondrial function and related genes expressions.

### Determination of Antioxidant Parameters

The activities of glutathione peroxidase (GSH-Px), superoxide dismutase (SOD), catalase (CAT), total antioxidant capacity (T-AOC), and the content of malondialdehyde (MDA) in serum, liver, and ileum mucosa were determined using commercial kits (Nanjing Jiancheng bioengineering Institute, Nanjing, China) according to the manufacturer's instructions. The total protein content in the liver and ileum mucosa was detected by the bicinchoninic acid (BCA) method. Each indicator was measured in duplicate simultaneously on the same plate.

### Isolation of Hepatic Mitochondria

Hepatic mitochondria were isolated using tissue mitochondria isolation kit by differential centrifugation (Beyotime Biotechnology Institute, Shanghai, China) according to the manufacturer's instructions. Approximately 150 mg of fresh tissue was minced with scissors and then placed in 1.5 ml of ice-cold isolation medium. The homogenate was centrifuged at 600 g for 5 min at 4°C, and the supernatant was separated and centrifuged at 11,000 g for 10 min at 4°C. The final mitochondrial pellet was resuspended in a storage buffer. The mitochondrial protein concentrations in the liver were determined by the BCA method.

### Measurement of Hepatic Mitochondrial Membrane Potential

The mitochondrial membrane potential was determined by a mitochondrial membrane potential assay kit with fluorescent dye JC-1 (Beyotime Biotechnology Institute, Shanghai, China). The isolated hepatic mitochondria were blended with the prepared JC-1 staining solution. Fluorescence intensity was measured with an automatic fluorescence microplate reader. The fluorescence value of JC-1 aggregate was detected at the 525 nm excitation and 590 nm emission wavelengths which was displayed as red fluorescence. The fluorescence value of the JC-1 monomer was detected at the 490 nm excitation and 530 nm emission wavelengths which was displayed as green fluorescence. Finally, the mitochondrial membrane potential was expressed as a red/green fluorescence intensity ratio (23).

### Determination of Mitochondrial DNA Copy Number

Total DNA was extracted from the liver and ileum mucosa samples using the *EasyPure*^®^ Genomic DNA Kit (TransGen Biotech Co., Ltd., Beijing, China) following the manufacturer's instructions. The DNA concentrations of the samples and the ratios of OD260/OD280 and OD260/OD230 were recorded. The samples were adjusted to the uniform concentration. RT-PCR reactions were performed on the CFX96 RT-PCR Detection System (Bio-Rad, Hercules, CA) with β*-actin* as the internal reference. Specific primers used in this study are listed in Table 2. The reaction steps were performed as follows: pre-denaturation at 94°C for 30 s, then denaturation at 94°C for 5 s followed by annealing extension at 57°C for 30 s in a total of 42 cycles of the program. The expressions levels of target genes relative to β*-actin* were calculated by the 2^−ΔΔCt^ method (24).

**Table 2:** Primer sequences for determination of mitochondrial DNA (mtDNA) copy number.

**Gene**	**Accession no**.	**Primer sequence (5^**′**^-3^**′**^)**	**Product size (bp)**
*mtD-loop*	MT800504.1	F: AGGACTACGGCTTGAAAAGC R: CATCTTGGCATCTTCAGTGCC	198
*β-actin*	L08165.1	F: GATTTCGAGCAGGAGATGGC R: GCCAATGGTGATGACCTGAC	90

*mtD-loop, mitochondrial DNA-loop*.

### Ribonucleic Acid Extraction and Real-Time Quantitative PCR

Total RNA in liver and ileum mucosa of broilers were extracted using the *TransZol* Up Plus RNA Kit (TransGen Biotech Co., Ltd, Beijing, China) according to the manufacturer's instructions. The purity and concentration of the isolated RNA samples were determined by Nanodrop ND-1000 (Nanodrop Technologies, Thermo Scientific, Wilmington, DE, USA) to detect that the OD260/OD280 ratios were between 1.8 and 2.0. After the quality control of RNA samples, the RNA was reverse-transcribed into cDNA using the EasyScript^®^ One-Step gDNA Removal and cDNA Synthesis Supermix Reverse-Transcription Kit (TransGen Biotech Co., Ltd., Beijing, China) according to the manufacturer's instructions. The following target genes: *Nrf2*, nuclear factor erythroid 2-related factor 2, *NQO1*, NAD(P)H quinone dehydrogenase 1, *HO-1*, heme oxygenase 1, *SOD1*, superoxide dismutase 1, *Gpx1*, glutathione peroxidase 1, *Nrf1*, nuclear respiratory factor 1, *PGC-1*α, peroxisome proliferative activated receptor gamma coactivator 1 alpha, *Cyt-c*, cytochrome c, *COX1*, cytochrome c oxidase subunit I, *COX5A*, cytochrome c oxidase subunit 5A, *SIRT1*, sirtuin 1, *TFAM*, transcription factor A mitochondria, were determined in this study. The primer sequences of the target gene are listed in Table 3. The reaction steps were performed as follows: pre-denaturation at 94°C for 30 s, then denaturation at 94°C for 5 s followed by annealing extension at 56–60°C for 30 s in a total of 42 cycles of the program. The expressions of target genes relative to β*-actin* were calculated by the 2^−ΔΔCt^ method.

**Table 3:** Primer sequences for real-time quantitative PCR analysis.

**Gene**	**Accession no**.	**Primer sequence (5^**′**^-3^**′**^)**	**Product size (bp)**
*Nrf2*	NM_205117.1	F: ATCACGAGCCCTGAAACCAA R: GGCTGCAAAATGCTGGAAAA	143
*NQO1*	NM_001277619.1	F: CCTCTACGCCATAGGGTTCA R: TGCAGTGGGAACTGGAAGAT	192
*HO-1*	NM_205344.1	F: GAAAGCTGCCCTGGAGAAAG R: CCCAGACAGGTCTCCCAAAT	175
*SOD1*	NM_205064.1	F: ATGTGACTGCAAAGGGAGGA R: AGCTAAACGAGGTCCAGCAT	176
*Gpx1*	NM_001329527.1	F: GACCAACCCGCAGTACATCA R: GAGGTGCGGGCTTTCCTTTA	204
*Nrf1*	NM_001030646.1	F: ACACAGCAACAGACCACAAC R: AACCTGGATGAGGGACACAG	108
*PGC-1α*	NM_001006457.1	F: CATGTGCAACCAGGACTCTGT R: ACGTCTAGTTCGGAGAGGTCA	111
*Cyt-c*	NM_001079478.1	F: AAGCACAAGACTGGACCCAA R: AAGAGAAGCCCTCAGCTTGT	70
*COX1*	NC_040970.1	F: CCCAAGCCCATGACCAATCT R: TGGAAGGTGCTTTCTCGGAC	168
*COX5A*	XM_003641804.4	F: CATCGATGCCTGGGAGCTAA R: CATTTAACCGTCTGCACGC	110
*SIRT1*	NM_001004767.1	F: AGTAGTAGCGAAAGCGGCTC R: TCGTTCCCTGCAGCTTCATT	194
*TFAM*	XM_015866188.1	F: GAAACGTGGCAAAATCTATCCG R: AGGTCTTCGCGTCCAAGCTC	131
*β-actin*	L08165.1	F: GATTTCGAGCAGGAGATGGC R: GCCAATGGTGATGACCTGAC	90

*Nrf2, nuclear factor erythroid-2-related factor 2; NQO1, NAD(P)H quinone dehydrogenase 1; HO-1, heme oxygenase 1; SOD1, superoxide dismutase 1; Gpx1, glutathione peroxidase 1; Nrf1, nuclear respiratory factor 1; PGC-1α, peroxisome proliferative activated receptor gamma coactivator 1 alpha; Cyt-c, cytochrome c; COX1, cytochrome c oxidase subunit I; COX5A, cytochrome c oxidase subunit 5A; SIRT1, sirtuin 1; TFAM, transcription factor A, mitochondrial*.

### Statistical Analysis

All data were verified to meet assumptions of normality and homogeneity of variance. The data were subjected to the Statistical Package for the Special Sciences (SPSS) statistical software (SPSS for Windows, version 25.0, Chicago, IL, USA) and were analyzed using one-way ANOVA. Significant differences were tested using the Duncan's multiple comparisons. All data are expressed as means ± standard error of the mean (SEM). Statistical significance was considered as *P* < 0.05.

## Results

### Antioxidant Parameters in Serum, Liver, and Ileum Mucosa

As shown in Table 4, acute heat stress increased MDA content and CAT activity in serum, GSH-Px, and CAT activities in the liver, and enhanced MDA, GSH-Px, SOD, CAT, and T-AOC in the ileum (*P* < 0.05). Compared with the HS group, TRP supplementation elevated SOD activity in serum and liver (*P* < 0.05).

**Table 4:** Effects of dietary tryptophan supplementation on antioxidant indexes in broilers subjected to acute heat stress.

**Item**	**TN**	**HS**	**HS+ TRP**	***p*-value**
**Serum**				
GSH-Px (U/mL)	749.41 ± 29.08	859.05 ± 40.07	824.64 ± 24.14	0.128
SOD (U/mL)	85.23 ± 3.49^b^	93.98 ± 1.65^b^	119.73 ± 3.95^a^	<0.001
CAT (U/mL)	4.56 ± 0.27^b^	5.64 ± 0.11^a^	4.14 ± 0.19^b^	<0.001
MDA (nmol/mL)	2.41 ± 0.20^b^	3.30 ± 0.18^a^	3.19 ± 0.34^a^	0.048
T-AOC (mM)	0.34 ± 0.03	0.30 ± 0.03	0.35 ± 0.03	0.538
**Liver**				
GSH-Px (U/mg prot)	32.98 ± 1.41^b^	45.36 ± 2.69^a^	36.51 ± 1.42^b^	0.001
SOD (U/mg prot)	458.81 ± 11.66^b^	448.98 ± 18.32^b^	521.88 ± 13.31^a^	0.012
CAT (U/mg prot)	55.16 ± 2.31^b^	65.40 ± 1.83^a^	59.62 ± 1.65^ab^	0.030
MDA (nmol/mg prot)	0.81 ± 0.11	0.99 ± 0.10	0.76 ± 0.10	0.304
T-AOC (U/mg prot)	1.00 ± 0.06	1.18 ± 0.04	1.08 ± 0.07	0.156
**Ileum**				
GSH-Px (U/mg prot)	19.57 ± 0.58^b^	29.84 ± 2.64^a^	25.52 ± 1.08^a^	0.006
SOD (U/mg prot)	101.52 ± 1.04^b^	119.42 ± 2.89^a^	128.15 ± 5.45^a^	0.002
CAT (U/mg prot)	2.87 ± 0.21^b^	4.26 ± 0.36^a^	2.70 ± 0.10^b^	0.006
MDA (nmol/mg prot)	0.25 ± 0.01^b^	0.35 ± 0.02^a^	0.28 ± 0.04^ab^	0.042
T-AOC (U/mg prot)	1.18 ± 0.06^b^	1.81 ± 0.16^a^	1.29 ± 0.03^b^	0.001

*Data are means ± standard error; n = 6 for each group. Mean values with different superscript letters were significantly different (P < 0.05). TN, thermoneutral group; HS, acute heat stress group; HS + TRP, acute heat stress + 0.18% tryptophan group. GSH-Px, glutathione peroxidase; SOD, superoxide dismutase; CAT, catalase; MDA, malondialdehyde; T-AOC, total antioxidant capacity*.

### Redox-Related Genes Expressions in Liver and Ileum Mucosa

**Figure 1:**
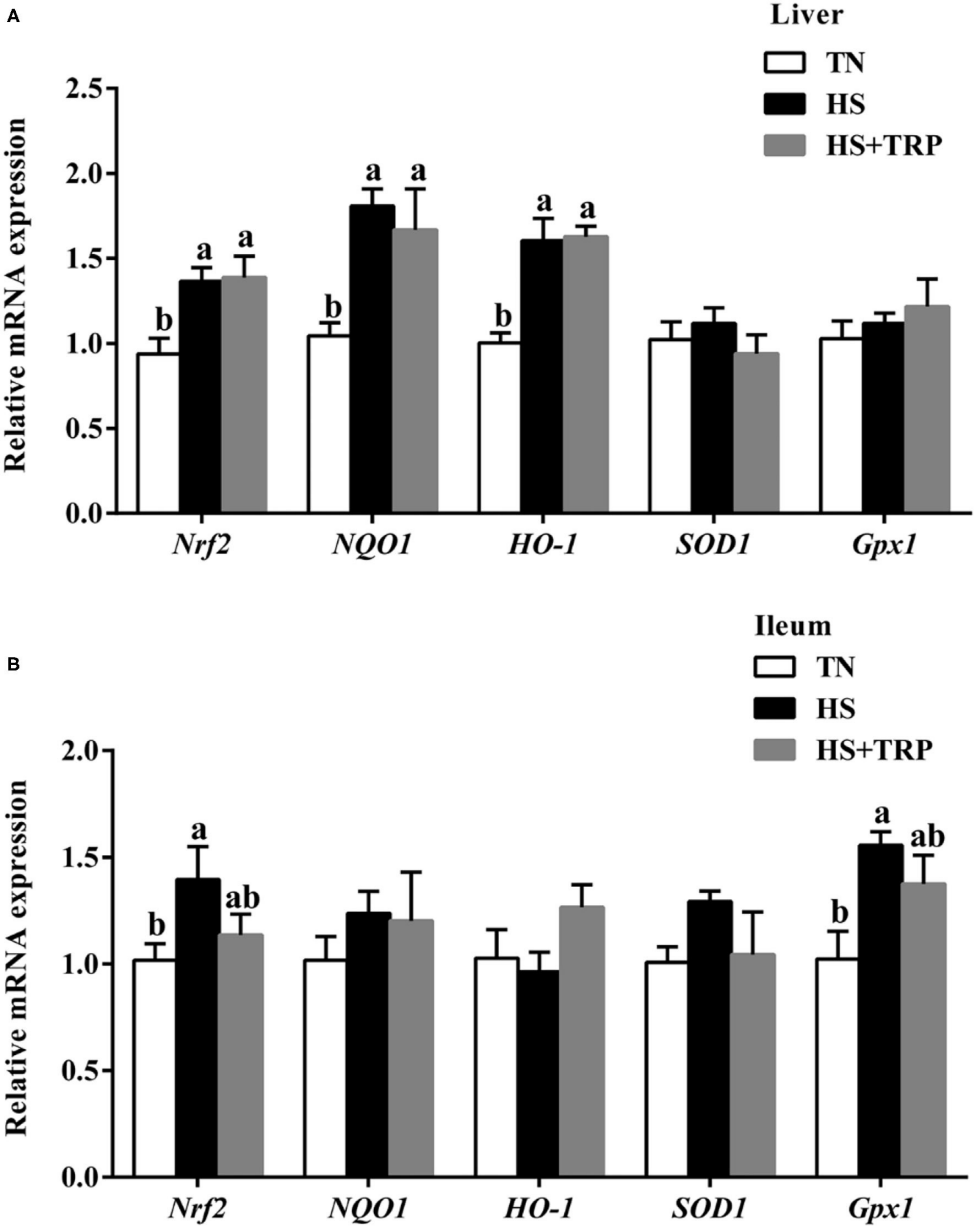
Effects of dietary tryptophan supplementation on mRNA expressions of redox-related genes in broilers subjected to acute heat stress. **(A)** In liver; **(B)** In ileum. Data are means ± standard error; *n* = 6 for each group. ^a,b^Different lowercase letters above bars indicate significant differences among treatments (*P* < 0.05). TN, thermoneutral group; HS, acute heat stress group; HS + TRP, acute heat stress + 0.18% tryptophan group. *Nrf2*, nuclear factor erythroid-2-related factor 2; *NQO1*, NAD(P)H quinone dehydrogenase 1; *HO-1*, heme oxygenase 1; *SOD1*, superoxide dismutase 1; *Gpx1*, glutathione peroxidase 1.

As presented in Figure 1, acute heat stress exposure led to higher levels of *Nrf2, NQO1*, and *HO-1* in the liver and *Nrf2* and *Gpx1* in the ileum as compared with the TN group (*P* < 0.05). Dietary TRP supplementation had no significant effect on redox-related genes expressions.

### Mitochondrial Membrane Potential in Liver

**Figure 2:**
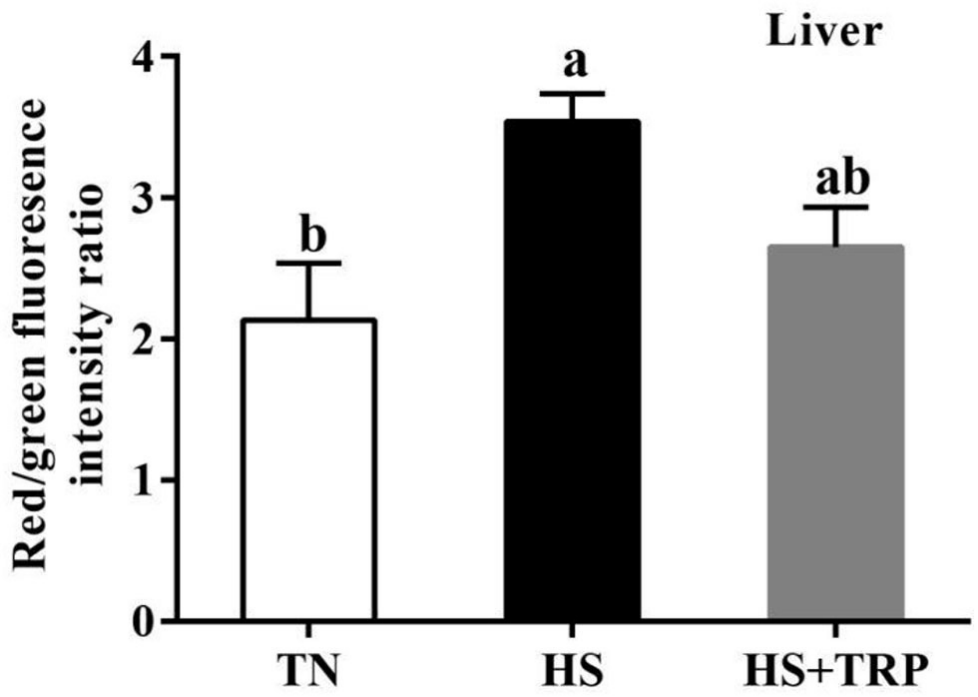
Effects of dietary tryptophan supplementation on mitochondrial membrane potential in liver of broilers subjected to acute heat stress. Data are means ± standard error; *n* = 6 for each group. ^a,b^Different lowercase letters above bars indicate significant differences among treatments (*P* < 0.05). TN, thermoneutral group; HS, acute heat stress group; HS + TRP, acute heat stress + 0.18% tryptophan group.

The mitochondrial membrane potential in the liver was greater (*P* < 0.05) in acute heat stress exposure broilers than that in the TN group (Figure 2). Compared with the HS group, mitochondrial membrane potential was not affected by TRP supplemented.

### *mtDNA* Copy Number in Liver and Ileum Mucosa

**Figure 3:**
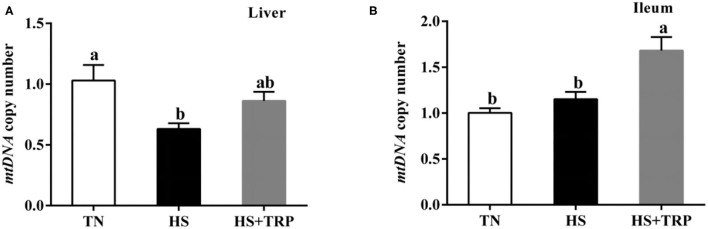
Effects of dietary tryptophan supplementation on *mtDNA* copy number in broilers subjected to acute heat stress. **(A)** In liver; **(B)** In ileum. Data are means ± standard error; *n* = 6 for each group. ^a,b^Different lowercase letters above bars indicate significant differences among treatments (*P* < 0.05). TN, thermoneutral group; HS, acute heat stress group; HS + TRP, acute heat stress + 0.18% tryptophan group. *mtDNA*, mitochondrial DNA.

Acute heat stress led to a reduced copy number of *mtDNA* in the liver as compared with the TN group (*P* < 0.05; Figure 3). This effect was abolished (*P* < 0.05) by TRP supplemented.

### Expressions of Genes Related to Mitochondrial Function in Liver and Ileum Mucosa

**Figure 4:**
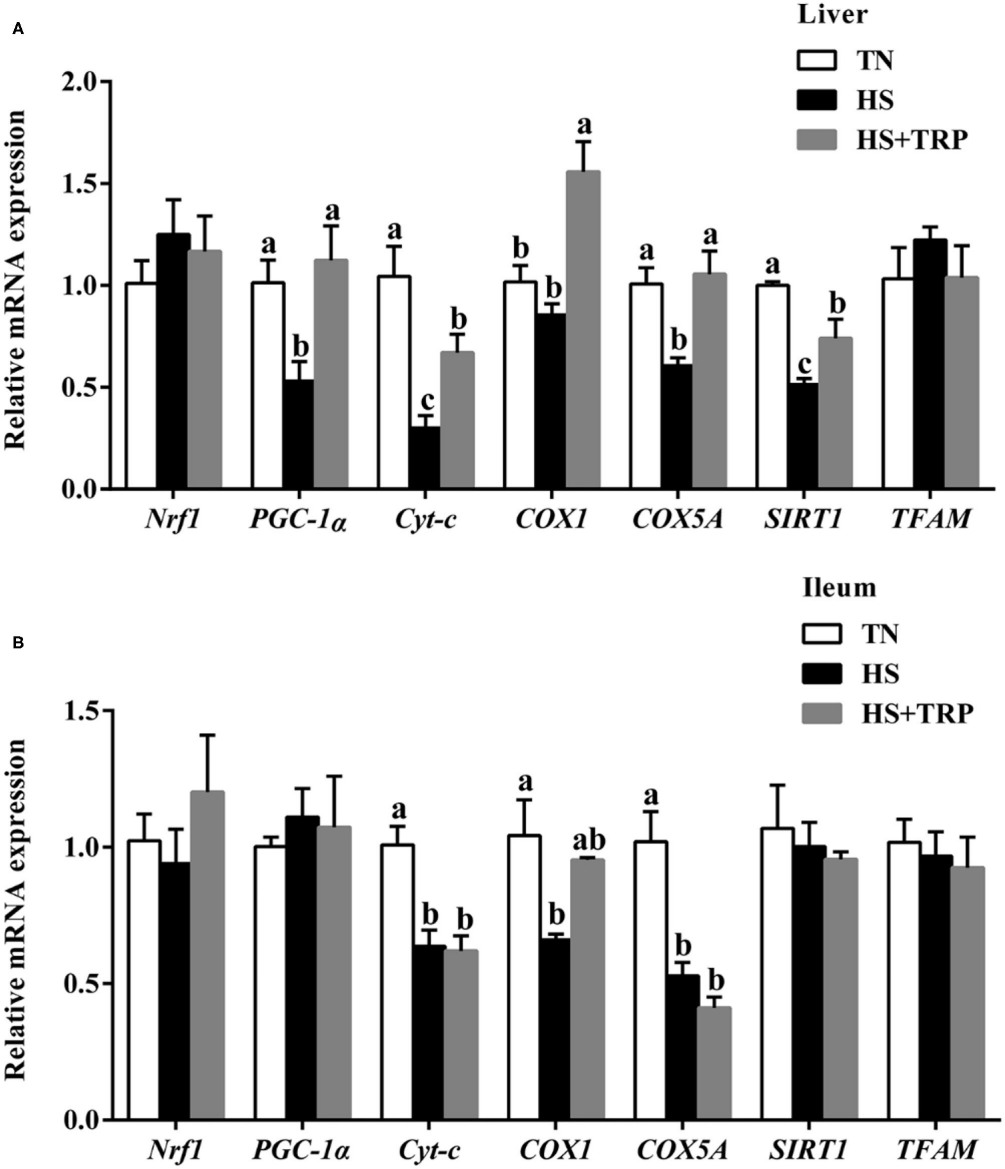
Effects of dietary tryptophan supplementation on mRNA expressions of mitochondrial function-related genes in broilers subjected to acute heat stress. **(A)** In liver; **(B)** In ileum. Data are means ± standard error; *n* = 6 for each group. ^a,b,c^Different lowercase letters above bars indicate significant differences among treatments (*P* < 0.05). TN, thermoneutral group; HS, acute heat stress group; HS + TRP, acute heat stress + 0.18% tryptophan group. *Nrf1*, nuclear respiratory factor 1; *PGC-1*α, peroxisome proliferative activated receptor gamma coactivator 1 alpha; *Cyt-c*, cytochrome c; *COX1*, cytochrome c oxidase subunit I; *COX5A*, cytochrome c oxidase subunit 5A; *SIRT1*, sirtuin 1; *TFAM*, transcription factor A, mitochondrial.

Compared with the TN group, the mRNA levels for *PGC-1*α, *Cyt-c, COX5A*, and *SIRT1* in the liver and *Cyt-c, COX1*, and *COX5A* in ileum were downregulated (*P* < 0.05) by acute heat stress exposure (Figure 4). The effects of acute heat stress on mitochondrial function-related genes expressions of *PGC-1*α, *Cyt-c, COX1, COX5A*, and *SIRT1* in the liver were reversed by TRP supplementation (*P* < 0.05).

## Discussion

Heat stress negatively affected the balance of antioxidant capacity and reactive oxygen species (ROS) production, which led to oxidative stress (8). The MDA is an indicator of oxidative stress representing the level of lipid peroxidation in the cellular (25). In this study, acute heat stress significantly increased the concentration of MDA in serum and ileum mucosa, which demonstrated caused oxidative damage to lipids, and in agreement with the previous study (26). The SOD, CAT, GSH-Px, and T-AOC are the key antioxidant enzymes and contribute to removing excessive ROS when subjected to the oxidative stress (27–29). Interestingly, this study observed the activities of GSH-Px, SOD, CAT, and T-AOC ileum mucosa, liver, or serum were increased by acute heat stress, which indicated acute heat stress may stimulate antioxidant defense system against oxidative damage. Of note, this work found that dietary TRP supplementation increased the SOD activity in broilers reared under acute heat stress. The structure of TRP contains a ring that can stabilize radicals through resonance or delocalization, which enables it to break radical chain reactions and exert antioxidant properties (30). However, the current results measured that TRP supplemented also reduced the activities of CAT, GSH-Px, and T-AOC in the broilers exposed to heat stress. Moreover, a previous study reported that dietary TRP supplementation had no significant effect on the MDA content and total antioxidant capacity in broilers subjected to high temperatures (31). The dosage of TRP, duration of heat stress, and the age of broilers may contribute to the discrepancy between this study and the previous study regarding the regulation of dietary TRP supplementation on antioxidant capacity, and the difference between enzyme activity and gene expression. Taken together, dietary TRP supplementation may regulate the oxidant–antioxidant balance in broilers subject to acute heat stress, but further studies are needed urgently.

Nuclear factor erythroid-2-related factor 2 (*Nrf2*) is an important transcription factor that regulates cellular oxidative stress and maintains intracellular redox homeostasis (32). A previous study observed that acute heat stress activated the *Nrf2* pathway (33). Consistently, this study confirmed that acute heat stress led to upregulate the mRNA expression of *Nrf2, NQO1, HO-1*, and *Gpx1*. These suggested that acute heat stress may activate the antioxidant pathway by increasing the expressions of *Nrf2* pathway-related genes in broilers. No differences were observed in the expression levels of *Nrf2* and its downstream genes including *NQO1, HO-1, SOD1*, and *Gpx1* between the HS and HS + TRP groups. It indicated that dietary TRP supplementation alleviates acute heat stress may not *via* the *Nrf2* pathway. Given the difference between antioxidant enzymes activities and gene expressions, it is reasonable to speculate that the *Nrf2* signaling and antioxidant enzymes are parts of the antioxidant defense system whose expression may depend on stress intensity and tissue specificity (34).

Mitochondria is the main site of energy supply and plays a crucial role in healthy function (8, 35). Notably, the mitochondrial membrane potential is an important indicator reflecting mitochondrial health status (11), a decrease of mitochondrial membrane potential is the hallmark event of early apoptosis (36, 37). In this study, acute heat stress markedly upregulated the mitochondrial membrane potential. The reason may be that acute heat stress increased the acceleration of energy consumption and activated mitochondria to produce more energy, and resulted in an increase in membrane potential. Consistently, previous studies observed similar results in birds, pigs, or other animals under stress conditions (38–41). Mitochondrial biosynthesis requires the coordinated function of the nuclear genome and mitochondrial genome (42). *PGC-1*α is a key transcription factor for mitochondrial biosynthesis and energy metabolism, *Nrf1* and *TFAM* are essential for mtDNA replication and transcription (43). *SIRT1* is the deacetylase that promotes mitochondrial biosynthesis and maintains mitochondrial function (44). *COX* and Cyt-c participate in the terminal reaction of the mitochondrial respiratory chain and affect ATP formation and the production and clearance of ROS (45, 46). In this study, acute heat stress significantly decreased the mRNA expressions of *PGC-1*α, *Cyt-c, COX5A, COX1*, and *SIRT1* in the liver and ileum. Consistently, our results observed that acute heat stress reduced the copy number of mitochondrial DNA in the liver, which was similar to the previous report (29). These results indicated that acute heat stress could cause mitochondrial dysfunction in broilers. Nicotinamide adenine dinucleotide (NAD^+^) is the metabolites of TRP, which plays a critical role in many enzymatic redox reactions and mitochondrial morphology, fitness, and function (47). It has been reported dietary TRP supplementation prevented piglets from compromising mitochondrial biogenesis processes and improved mitochondrial function (23). Indeed, this study found that TRP supplementation increased the mRNA expressions of *PGC-1*α, *Cyt-c, COX1, COX5A*, and *SIRT1* in liver of broilers reared under acute heat stress. Thus, these suggested that the protective effect of TRP might be associated with protecting mitochondrial function in broilers subjected to heat stress.

## Conclusion

In summary, it is concluded that dietary TRP supplementation could regulate the oxidant–antioxidant balance and alleviate mitochondrial dysfunctions in acute heat-stressed broilers. This study demonstrates that the supplementation of TRP in diets might be an effective nutritional strategy to protect against acute heat stress impairment.

## Data Availability Statement

The original contributions presented in the study are included in the article/supplementary material, further inquiries can be directed to the corresponding author.

## Ethics Statement

The animal study was reviewed and approved by Laboratory Animals Ethics Committee of Jiangxi Agricultural University, Nanchang, Jiangxi, China.

## Author Contributions

GL and JO conceived and designed the experiments. JO, QL, JZ, CC, and SG collected samples and performed the experiments. JO, JY, and HZ performed the analysis. JO and HZ wrote the manuscript. GL and HZ directed the analyses and revised the manuscript. All authors reviewed and approved the final manuscript.

## Funding

This work was supported by the National Natural Science Foundation of China (Grant No. 31860651) and the Key Project of the Natural Science Foundation of Jiangxi Province (Grant No. 20181ACB20015).

## Conflict of Interest

The authors declare that the research was conducted in the absence of any commercial or financial relationships that could be construed as a potential conflict of interest.

## Publisher's Note

All claims expressed in this article are solely those of the authors and do not necessarily represent those of their affiliated organizations, or those of the publisher, the editors and the reviewers. Any product that may be evaluated in this article, or claim that may be made by its manufacturer, is not guaranteed or endorsed by the publisher.
